# Family Carers of People with Young-Onset Dementia: Their Experiences with the Supporter Service

**DOI:** 10.3390/geriatrics1040028

**Published:** 2016-11-05

**Authors:** Aud Johannessen, Knut Engedal, Kirsten Thorsen

**Affiliations:** 1 Norwegian National Advisory Unit on Ageing and Health, Vestfold Hospital Trust P.O. Box 2136 NO-3103 Tonsberg, Norway; knut.engedal@aldringoghelse.no (K.E.); kirsten.thorsen@nova.hioa.no (K.T.); 2 Norwegian Social Research (NOVA), University College of Oslo and Akershus, Oslo 0001, Norway

**Keywords:** accessibility, caregivers, early onset dementia, public health, qualitative study, respite care, volunteers

## Abstract

Background: Family carers and people with young-onset dementia (YOD) require tailored assistance as dementia progresses. A variety of health care services is needed, including supporter services. To our knowledge, research focusing on experiences with the supporter service is scarce. Aim: To evaluate the supporter service by examining how primary family carers experience the assistance provided. Method: Qualitative interviews with 16 primary family carers of people with YOD were performed from 2014 to 2015. Content analysis was used to analyze the data. Results: Three main themes emerged from the interviews. First, a good match focused on the carers’ experiences of the relationship between the supporter and the person with YOD and included three subthemes: a nice, empathetic personality, a friendship-like relationship, and the content of the meetings. The second theme, relief, addressed the carers’ experiences with the service. The third, coordination, concerned the carers’ relationship with the health care service. Conclusion: Developing tailored services and assistance initiatives is important. A well-organized supporter service is a valuable supplement to formal programs and should be developed as part of an overall support package.

## 1. Introduction

Dementia is a syndrome that can be caused by a variety of brain disorders. A common element is that most brain disorders that cause dementia gradually lead to a decline in cognitive and adaptive functioning, resulting in problems with memory, recognition, and reasoning [1]. This leads to increasing helplessness and impairment in activities of daily living and to more caring tasks for the family of the person with dementia [2,3]. In addition, the primary family carer as well as the person with dementia may become socially isolated. This isolation may pose an increasing burden on the carers as well as the person with dementia over time [1,2,3,4,5].

Dementia mainly occurs in older people, but it can also develop in people younger than 65 years of age [6,7]. It is estimated that approximately five percent of all people with dementia are younger than 65 years, which means that in Norway, at least 4500 people have dementia before the age of 65, based on estimates by Zhu and colleagues [7]. People with dementia at this age and younger are defined as having young-onset dementia (YOD).

The lives of people with YOD generally differ from those of older people with dementia, as people with YOD might have children living at home, be physically fit, and be part of the workforce. These differences generally mean that people with YOD and their families have needs that differ from those of their older counterparts [8,9,10,11,12,13,14]. Caring for people with YOD has a significant impact on partners and other family carers, as they typically gradually assume more caregiving tasks and experience social stigma, increased anxiety and depression, and reduced quality of life [15,16]. The impact on family carers may continue during the long course of illness [1,16] and usually increases as dementia progresses.

Assistance during the course of dementia such as day care, respite care in institutions, and care from non-professional and professional instances can be helpful for these families, as described in the Norwegian government’s 2020 dementia care plan [17]. Assistance from supporters (in Norwegian: support contacts) is listed in the Norwegian health care legislation as one of the possible services [18]. In the legislation, a supporter is described as one who “helps another person with social contacts and activities. The supporter shall help him/her to be more self-confident, to cope better with different situations in life and to get into contact with other people. The supporter can visit at home and accompany the person with dementia to the cinema, sports events, and other social activities.” The service aims to provide a social life and meaningful leisure time for people who need assistance. Supporters can be provided individually or in groups. Supporters are thus primarily meant for the person with dementia, aiming to address his/her varying and individualized needs, which change as dementia progresses.

In general, supporters are not commonly used in Norwegian dementia care, and the hours allotted for this service are usually small. Only two-thirds of the Norwegian municipalities offer a supporter service for people with dementia or their carers [19]. This service may influence the ability of the carers as well as the people with dementia to participate in everyday life and maintain a level of participation in the workforce and society.

A few specialized care models exist for people with YOD in Norway [16,19], such as nursing homes, day care centers, and some organized activities designed for people with YOD. To our knowledge, tailored services to support people with YOD and their primary family carers are rare. Organizing these types of tailored services for families with YOD can be demanding, since the disorder is rare in this age group. Although people with YOD do not represent a large group, those affected and their families encounter significant demands and problems. For that reason, the supporter service might be one of several services that could be beneficial for families as well as for people with YOD [4].

The supporter service is the equivalent to what is called respite carers or volunteer workers in other Western countries, although the organization of the service may differ. Supporters in Norway are assigned to a person after an application based on the person’s needs is accepted. They are employed and paid by the municipalities for a certain number of hours a week, while the expenses for the activities are covered by the person. Supporters must ideally possess adequate competence and skills to work as supporters, but in practice, there are no requirements regarding special education or preparatory courses. Therefore, practices within supporter services may vary between municipalities [20,21]. The support service in Norway is thus structurally positioned somewhere in between public, private, and voluntary; formal and informal; educated and unskilled; specialized and unspecialized; and rigid and flexible services.

Research focusing on the supporter service is rare. Based on these findings, we conducted the present study to evaluate the supporter service in Norway by examining how primary family carers experience such assistance.

## 2. Methods

### 2.1. Design

A qualitative study design with in-depth interviews was selected to describe the participants’ experiences with the supporter service. Manifest qualitative content analysis was adopted as a relevant and effective method to analyze the data obtained in the study [22].

To obtain heterogeneity, we asked 17 primary family carers of people with YOD who had been assigned a supporter to participate. They were recruited from four memory clinics in three municipalities and came from seven different municipalities. All carers and people with YOD had been informed about the diagnosis of dementia by a psychiatrist or geriatrician. One family carer declined participation; we thus interviewed 16 family carers. Characteristics of the informants and the person with YOD are described in Table 1.

The participants included two wives, five husbands, two partners, one sister, one brother, four daughters, and one son. Five of the people with YOD resided in nursing homes, three had short-term stays in a nursing home while still living at home, seven were sharing households with spouses or partners, none stayed with a daughter, son, sister or brother, and three lived alone.

### 2.2. The Interviews

The data were collected through individual interviews. The interviews took place in 2014 and 2015 at the most convenient place for the informants and were conducted by the first author in accordance with qualitative interviewing methodology outlined by Kvale [23,24]. Twelve of the interviews took place in the informants’ homes, three at the interviewer’s workplace, and one took place in a group room at a hotel. The interviews lasted between 15 and 79 (mean = 30) minutes. The interviews were tape-recorded and then transcribed verbatim by a professional typist within two weeks. A quality control check of the transcripts was performed by the interviewer by listening to the tapes while reading the transcribed interviews.

The interviews were based on a guide with two main open-ended thematic questions that focused on the family carers’ experiences with the supporter service offered to the person with YOD. The main questions asked were, “Can you tell me about your experiences with the supporter service?” and “Is the service beneficial for you and your family member with YOD?” Depending on their replies, the aspects and ideas raised by the informants led to further questions to obtain additional information. The interviews also obtained biographical information about the partner, the diagnosis, the progression of dementia, and experiences with the supporter service.

### 2.3. Analysis

The transcriptions of the data were analyzed using qualitative content analysis as outlined by Graneheim and Lundman [22]. Initially, the transcribed text was read carefully several times to establish an overall impression. Then, “meaning units”, i.e., words and sentences expressing a significant meaning related to the research questions, were identified. These units were later systematically condensed. In the second stage, the condensed meaning units were labelled with “a code”, indicating their content. In the third and final stage, the main themes at a higher analytical level were identified. The themes were based on related groups of codes. Special attention was paid to the differences between and similarities among the themes and subthemes [22]. The main themes indicated the core aspects of the experiences with the supporter service. A.J. and K.T. were primarily responsible for the analysis, but the process was continuously discussed with the third author.

### 2.4. Ethics

The present study followed the ethical principles outlined in the Helsinki Declaration [25]. The study procedures were presented to the Regional Committee for Ethics in Medical Research, Southern Norway, and were subsequently approved. Consent from the informants was obtained after they had received oral and written information and before the interviews took place.

## 3. Results

The results are presented by the analytical themes based on the narratives of the carers, focusing on their experiences and views. The main theme of the interviews pertained to their experiences of how the supporter service influenced the well-being of the family member with YOD, whose reactions greatly influenced the everyday life of the carers. If the person with YOD was dissatisfied with the supporter service, then the carer was deeply concerned, worried, and dissatisfied. The carers also evaluated the service in terms of their own needs, but most of the reactions were intrinsically intertwined with the experiences of the person with YOD. A remark from a husband illustrates the interdependent reactions to the supporter service. “I myself think that it (the supporter service) was very positive, and just that my wife is looking forward to something positive; it cheers her up”.

As expected, some informants were more informative, were closer to the service, had more experience, and provided richer descriptions. A few stories are presented in greater detail to expose specific analytical points. When the meaning remained clear, the term “supporter service” was shortened to “service”. The results are condensed into three main themes, highlighting different aspects of the service.

The first theme, a good match, focused on the relationship between the supporter and the person with YOD. The analysis of this theme resulted in three subthemes: “a nice empathetic personality”, “a friendship-like relationship”, and “the content of the meetings”. The second theme, relief, focused on the family carers’ experiences with the supporter service in relation to his or her personal life sphere, interests, and needs. The third theme, coordination, pertained to the carers’ relationship with the health care service.

### 3.1. A Good Match

#### 3.1.1. A Nice Empathetic Personality

When the family carers described the qualities of the supporter service, they emphasized the personal qualities and characteristics of the supporter through descriptions of their personalities. “These two [supporters] she now has are really fantastic people who have good insight and understanding. Yes, really warm and good people. Therefore, this (service) has been very good for her”. Warm, nice, good, kind, and cheerful were repeated adjectives describing the appreciated supporters, as were empathetic and understanding. The importance of personal qualifications is highlighted in this excerpt: “I think it is more decisive who [the supporter] is and their personality (…). Yes, that they are calm, infer trust and…Yes, I think that is more important than age”.

Other characteristics of what defined “good supporters” were being patient, calm, and secure. Some carers mentioned experience and maturity. A long and varied life was also mentioned by some, as this experience was assumed to lead to more coping abilities. “These two supporters were elderly women who had a lot of patience. They had been out in a cold winter earlier in life, and they were in a way very calm and secure in themselves and their role, really. They did not become alarmed when Mom sometimes did strange things; they coped very well with the situation. Mom was very fond of them”.

The personality of the supporters should also match the person with YOD. “It was rather accidently that it became her. However, I feel that she quickly felt a sort of concern, a positive chemistry for Mom—that she felt that the two of them had a nice way of understanding each other. Therefore, she felt that the task was rather interesting, and she had never been a supporter before. Mom was very fond of her, a perfect match at once”. Here, we see that mutual liking and understanding could be decisive for recruitment of or enlisting a supporter. Others may gradually reach a “good match”.

A highly valued personality characteristic was *flexibility*, the ability of the supporters to accommodate their reactions, schedules, and activities to the needs, interests, and moods of the person with YOD. As one person stated, “They were people who were flexible. I think that was the best part of the experience, that they were very flexible. They could rearrange things. Suddenly when something interesting was coming up, they just said, ‘Okay, then we will drop it on Thursday and do it tomorrow instead.”

A few had some negative experiences with the supporters. The negative characteristics mentioned were unreliability, inflexibility, lack of energy, inability to adapt to the progression of dementia, or simply that they were not a good match. One wife mentioned that several people were sent to them from the administration without any regard for matching of interests. Additionally, there were supporters who were uninterested or unfit for the task. The administration did not seem to prepare them for the job. One elderly supporter did not know anything about dementia; another suddenly disappeared after a few meetings; and some young people rarely found the time to visit or did not show up at the agreed upon time. “They seemed barely able to take care of themselves”, as one carer remarked. When the supporter was unfit, unreliable, or a mismatch, it was the responsibility of the family carers to observe, react, and readjust to obtain better service. The family care became responsible for monitoring the match. The core aspect of the carers’ experiences of a good supporter service was then summarized by the qualities of a “good human being”.

Questions about other characteristics of the supporter, gender and age, were touched upon. All the current supporters were of the same gender as the people with YOD, except for one man who had a woman as a supporter; however, the ages of the supporters varied. Some informants mentioned that age and gender were not decisive, as was personality, but some indirect consequences of age and gender did matter. They indicated that an older age of the supporter may increase maturity, imply varied experiences with people of all ages, support self-confidence, enhance coping abilities and relational competency, and offer a better match of shared life experiences. One woman who had negative experiences with young supporters for her husband underlined the value of both age and gender. “He needs an adult person, probably a man, who he can do adult-boy things with”, gender may influence common interests and activities. Both age and gender could be important for shared understanding. These factors are also significant in forming friendships.

#### 3.1.2. A Friendship-Like Relationship

Many of the family carers described the relationship between the supporter and the person with YOD using concepts from the informal and personal spheres, addressing what constitutes friendships. The informants accentuated aspects such as openness in conversations and feelings of trust as well as a mutual understanding and fondness for one another. The supporter and person with YOD may also find some sort of equality. A daughter reflected on the relationship: “It seems as—maybe it is wrong to use the word—a friendship relationship (…). She (the supporter) shares information about her own family, and Mother about hers. She is in a way not there as health personnel of any sort. She is more a human being. I think that is a very positive part. They are having real conversations that are not constructed in any way. Mother has calm talks with the supporter. I think it fills in some shortcomings (in the health services).”

The daughter underlines that their contact is based on a relationship with “real personal talks” not specialized, professional conversations. She felt that this was lacking in the mainly short visits conducted by many different nurses. She continued her reflections: “For if you only meet people who come with pills or give you practical help, then you do not have the social part there. A practical helper who washes or nurses who focus on medicines and supervising health status do not provide that sort of relationship.”

This participant—similar to the other informants—valued the stability of the supporter, the relationship, and the fact that the same person visits. The relationship could then become well-known, predictable and safe. “It is an advantage that it is the same (person), not new people all the time.” For a person with progressing dementia, stability and predictability are important.

#### 3.1.3. The Content of the Meetings

Some of the meetings between the supporter and the person with YOD focused on one activity, whereas others involved a wide variety of events and leisure activities. These meetings mostly occurred outside of the home as visits to public places for “normal” everyday activities. A main aim of the outings, as the carers perceived it, was to introduce variation into everyday life, outside of the family realm. “For otherwise, her days would be very much the same and monotonous” as one daughter remarked.

Even if the supporter usually stays at home with the person with YOD, the visits provided valuable breaks in daily life. The carers underlined the importance of having something to look forward to. The visits created a structure to the weeks. The activities were usually adapted to the interests of the person with YOD. As people with YOD are middle-aged, they are often still physically fit and have a need for physical exercise and activities, which are often neglected in the public health service system. “Yes, she is very fond of walking, so that was the starting point. Therefore, she and the two supporters go out walking nearly every day, for shorter or longer strolls, depending on the weather and their energy”.

Some supporters continued their activities after the person with YOD had moved into a nursing home, where such activities were often at a minimum. One man, now living in a nursing home, had a male supporter who had provided support while the man was still at home. The supporter had helped him go on walks and take trips by car. He, the supporter, and two other retired men had formed “a buddy-group”, in which they visited “special places”. In this manner, they all enjoyed the trips and shared the positive aspects of the service: variations in daily life, events to look forward to, physical activity, and masculine relationships. The outings thus served multiple functions, not the least of which was bolstering their younger masculine identity, an especially valuable benefit in predominantly female settings, such as in nursing homes.

Many carers reported engaging in varied activities, often ones in which the person had participated earlier in life. “She likes to travel around, drink coffee, and stroll along. Yes, we travel around, visit cultural events, and do some shopping, and we enjoy just sitting around with a cup of coffee and looking at people.”

Some informants discussed the meetings with the supporter at home. A daughter said the following: “Then, they have evenings when they sit together for hours knitting, and she (the supporter) is learning knitting techniques from Mammy.” The supporter in question was a woman from Eastern Europe. Knitting is an example of a typically female activity, in which women with YOD may still maintain competence. The woman with dementia assumed a role in which she was the expert and had specialized skills to teach. This social situation presented a normal gathering, based on shared interests, mutual liking, and equality, with an advanced performance of the woman with dementia. The situation and relationship bolstered her self-image and self-confidence, as recounted by her daughter. At other times, the helping relationship shifted the other direction. “When Christmas approached, she (the supporter) was there and helped mother with wrapping gifts and different things.”

We only met one carer who was rather dissatisfied with the activities as well as with the supporter they currently had. One daughter recounted that her father, now living in a nursing home, had a male supporter who took him bowling two times a week (three hours each visit). “They do the same thing all the time. However, this is the thing my father likes, so it is rather difficult”. Despite those misgivings, she admitted that it is “a good thing to have someone to take him out”.

Over time, relationships with supporters can change for many reasons. The situation of the person with dementia may even improve, which at times is attributed to the supporter service. One daughter described a positive transformation in her mother’s everyday life: “I think her everyday life in general has become better in many ways. For many years, Mother did not watch TV and did not read anything. In addition, now she watches TV series. Also, she knows that the supporter comes on Fridays, so she looks forward to that, and afterwards she ‘lives a bit’ on that. She has someone visiting her. I think that is an important part.” The daughter summarized her mother’s and her own experiences: “It is entirely positive.”

The relationship between supporter and person with YOD can also change as the dementia progresses. We received narratives about supporters who were able to adapt to the development of the dementia very well because they knew the person in his or her more well-functioning days. “The service did function very well. Mom still had language abilities then, and she was still in such a stage that she could see that she was ill (…), could see herself from the outside. So, the supporter understood what Mom said, because she had been a part of the process early. Therefore, they had a very nice time together. The supporter also adjusted her management to the development of the disease. Later, Mom had to be protected from herself. She had to be shielded because she began to say and do so many strange things. The supporter had a lot of patience.”

For others, the supporters’ tasks may have eventually become too difficult to manage; the activities could lose their meaning and attraction. The closeness could become too emotionally exhausting. One daughter stated, “The last supporter, she became so close to Mom, for good and for bad (…). In addition, she has been at the office for home services and cried and wished that someone else would take over.”

Over time, supporters could change plans for other reasons. Some seemed to have lost their dedication. One of them felt “empty”, as a family carer reported. Often the contact ended when the person with YOD moved into a nursing home.

#### 3.1.4. Relief

The theme relief encompasses several aspects. Emotionally, it implies more relaxation and peace of mind for the family carer, as they could enjoy less worry and stress for a while: “When the supporters are there, then there is no problem. Then, I relax”, said a daughter. The service allowed the family member to have more time for themselves.

The supporters were also important regarding the working situation of the family carer. Visits and meaningful activities reduced the carers’ worries about the person with YOD having empty, lonely, passive days and their anxiety over the fact that the person with YOD could venture out without a companion. The carers reported that these worries and interruptions interfered with their working capacity. Experiencing relief may thus be vital for them to concentrate on their job. A husband recounted how he had to interrupt business trips and meetings at work several times to take care of his wife. He felt relief when the administration allowed a more flexible coordinated solution for the supporter service that was better adapted to his working schedule. This sense of relief could even determine the willingness of the carer to continue caring for the person at home. Another husband said, “now it (the supporter) is a fundamental requirement for me to be able to have her at home. She has much anxiety and agitation. She calms down when she does other things that direct her attention away from being at home.”

When the service was functioning well, as most of them were, what the carers wished for the most was extended hours. “So, there is really no negative thing to say. The only thing may be that there should be more time.” One husband remarked, “It is fishing that tops my list of activities in summertime. Earlier, I could go away on weekends and go fishing. This is perhaps what I miss the most.” The family carers wanted more time for themselves and to fulfil their own interests. They valued opportunities to have more contact with their own children, grandchildren, other family members, and friends, without having caring obligations.

#### 3.1.5. Coordination

All the informants were involved in the families’ coordination of the supporter service. Their experiences with health care administrations varied greatly, from smooth, supportive well-organized relationships (after the diagnosis was established), to disengaged contact and even rigid, impersonal, and inflexible relationships. Before a diagnosis had been determined, the family carers usually found it very difficult to receive assistance with the care.

In some cases, service administrators created a total package of different types of assistance—practical and medical in-home help, day care centers and supporter service. They were able to rearrange the combination of these resources as the person’s dementia progressed. The administrators were easy to contact and converse with, and they followed the families over time. “The supporter is just a piece of the package.”

Family carers appreciated when the administrative personnel were open-minded and flexible and allowed the supporters to be flexible in their dialogue with the person with YOD and, if necessary, with the carers. “That is, not just Monday or Thursday from 6:30 to 8:00 p.m., but it is the number of hours a month that they can do this. For there can be weeks when she has more needs than other times. So, the support is also flexible and has the ability to be flexible. That is very good”.

They valued agreement on the purpose of the service. “Yes, we had a very good dialogue, and we only focused on the fact that Mother should have positive experiences, nice moments, especially feel joy over songs, music, and such things”. When the supporters were allowed to reorganize time and content together with the person with YOD, it lessens the administrative burden for the family carers, which was a relief. Some supporters had added enough hours for the carer to stay away for a night or a weekend trip. One carer found that the administration had changed the hours established for home nurses to the supporter service “so that there were not too many people visiting her.”

Others reported rather negative experiences with the administration. One husband said that he had initially received a message that the supporter had to be a person with whom they were not related and that the service could only be used for cultural activities. He was working full time and was worried because his wife was sitting alone for much of the day. They had tried nurses who came to their house, but according to his wife, more than 20 people were visiting her at different times, some only to see “that she was on her feet; others came for five minutes. She became completely confused, and in the end, she refused to accept home nurses.” They were then provided four hours a week with the supporter service and attendance at a day care center five days a week. His wife no longer had enough energy to go to the cinema, etc. He had managed with substantial efforts to persuade the administration to allow the supporter to visit his wife at home, “so that she gets some stimulation from people other than just me. Then, I can go out walking and visit the cinema”.

## 4. Discussion

This study explores the narrated experiences of carers of people with YOD with the supporter service in Norway. People with YOD are diagnosed with dementia rather early in life, at a time when most of them have families, partners, and sometimes also children living at home, and family members foresee a normal life course and ageing process [11]. Due to the extraordinary circumstances and the relatively small number of people with YOD, the health care system has tended to overlook the problems and neglected the needs of both people with YOD and their families [16,19,21,26]. To develop tailored day care services and nursing homes to provide care for such a relatively small group of people is demanding. Therefore, it is necessary to better understand family carers’ experiences with the supporter service to be able to offer improved assistance. In Norway, the supporter service is integrated into the public health and social system and the municipalities pay for, regulate, and determine the hours assigned to the users.

We found that the experiences of the family carers were closely interrelated with those of people with YOD and were deeply influenced by how well the assistance met the needs of the person with YOD. As shown, the experiences of the family carers varied from high levels of satisfaction to frustrated disappointment with a service maladapted to the needs of both the person with YOD and the carers. However, most of the carers were very satisfied. An unsatisfactory supporter service had usually been replaced by the time the interviews took place.

Additional results of the present study are discussed according to the organization of the main themes and subthemes. We found that a successful supporter service as perceived by the family carers basically depended on having a good match between the supporter and both the person with YOD and the family carer. Several participants had privately recruited supporters through families and friends, which often implied “a good match” as well as engaged and motivated supporters. The fact that the people knew each other facilitated the establishment of the service. Continuity theory [27] underlines the importance of outer and inner continuity to adapt to changes during aging, in which continuity links experiences to a person’s life history. Continuity becomes especially important in life phases with major changes, such as when dementia progresses. Additionally, family members experience substantial changes during the progression of dementia and have a need to continue their own life activities; the supporter service may provide valuable assistance in this regard. One study noted that the potential to build good relationships with family carers was a factor that encouraged dementia care supporters to continue their work [20].

One of the most fundamental aspects of a successful supporter service for the person with YOD was the quality and personality of the supporter. The informants described the supporters as being nice, kind, patient, experienced, mature, and empathetic, and they were able to cheer up the person with YOD. Some carers emphasized that mature supporters were familiar with both the positive and the darker sides of life. The supporters were tolerant of variations in behavior and were able to cope with the symptoms of dementia when the behavior became challenging or embarrassing. The supporters were able to assist the person with YOD cope with stressful and challenging situations. The coping tasks required of people with YOD and their carers are challenging, and they need both formal and informal support and assistance [26,28]. Supporters who were deemed unfit for the service by the family carers either did not understand or were not able to adapt to the signs of dementia, and some could not bear the deterioration in the person’s condition.

One factor mentioned or implied in the narratives as being relevant for good relationships was the gender of the supporter. Having the same gender made it easier for the supporter to identify engaging activities for the person with YOD. Age did not seem to matter as much and was considered secondary to personal qualities. There were stories about a well-functioning supporter service provided by both younger and older generations. Older supporters or pensioners had the advantage of having more spare time and the ability to practise more flexibility.

The supporter was a friendly visitor who came regularly over time. Typically, contact with friends and acquaintances decreases as dementia progresses, and the condition infers social isolation for the person and the family carer. The supporter provided valuable friendship-like social contact. According to Amichai-Hamburger and colleagues [29], what distinguishes friends from acquaintances is, first of all, intimacy or closeness. The second characteristic is companionship, i.e., enjoying spending good times together. Third, friends provide support—emotional, social, and material—in times of stress and need. Several studies also indicate that similarity is important in characterizing friends [29,30,31,32]. Some informants mentioned that the interactions between the supporter and the person with YOD were based on certain forms of equality in participation and communication, and this equality was greater than that in contacts with professional personnel.

The foundation of the carers’ relationship with a well-functioning supporter was trust [16]. When the family carer trusted the supporter and they communicated well, everyday life and administrative tasks were easier. Decisions could be delegated. Trust also encompassed a carer’s confidence that the supporter could cope with difficult situations and behavior.

Personally, the basis of a well-functioning supporter service for the family carer was that it provided relief in many ways: emotionally, allowing for fewer worries; practically, facilitating everyday life and working situations; and personally, providing some free time to pursue personal interests. These findings are consistent with other studies [16,28,33]. Caregiving at home could be extended when receiving the supporter service, or as one husband said, overall care could eventually be coordinated using a total package of varied support measures. The emotional stress and burden experienced by family carers of people with YOD are exhaustive and undermine their normal everyday life, work, family life, and social networks [11,16]. The later stages are considered a “torment” [16]. Our study shows that the supporter service offers substantial relief.

An important aspect of the supporter service is that it is “in-between” the formal and informal systems; it is formally administered under the health service, is paid and regulated, and provides personal and individual contact with the person with YOD. What the family carers underlined as important was that the supporter service improved the experience of normality and ordinary everyday life for the person with YOD. To “get out of the house”, attend events and cafes, go shopping, participate in local life, or have personal visits at home are all activities that continue one’s participation in society. By supporting some of the abilities that the person with YOD still may have, the supporter service bolstered the person’s sense of self and self-confidence. The service focused on the person, not the disease. Supporting “personhood” is emphasized in a study by Tolhurst and colleagues [34]. The supporter service functions as a bridge to a person’s former life and provides a sense of coherence [35]. Being respected and treated as a human being in normal everyday situations is vital to a person’s well-being and quality of life. Family members of people with YOD need assistance in these tasks [8,10,11,33].

The third theme, coordination, focused on the importance of having a “smooth” and understanding relationship with the municipality administration and maintaining good dialogue or communication in which the needs of the family carers were also taken into consideration. As demonstrated, flexibility is crucial for a well-functioning supporter service according to the family carers’ experiences. This construct encompassed both flexibility of the supporter to adapt to the situation of the person with YOD and flexibility of the administration to tailor the support to the needs of the person with YOD and their family carers. The family carers greatly appreciated the well-planned and coordinated support service, organized by a dementia team or a memory clinic. This level of supporter service could become “the glue” in the total package of services and in the everyday life of families of people with YOD, as noted by another study [33].

This study documented the aspects of the supporter service that the family carers valued, factors that the administration should use as a foundation for planning and implementing the service. As dementia develops, the supporters may need contact with professionals to discuss emerging problems and obtain advice. In accordance with other studies [36,37], supporters should be informed and supported while maintaining the informality of the contact with the people with YOD. In this way, the supporter service should be more systematically integrated into the health care system, coordinated, and expanded. Another study emphasizes that organizational structures must have administrators who can provide guidance and education to the supporters, improve the quality of services and facilitate the recruitment of supporters [21]. We found that what the carers wanted the most was a more supporter service. This service is rarely used in dementia care [19]; the supporter service is perceived as filling “a gap” in the public services. The “gap” especially affects younger people with dementia and their carers, who are more actively involved in working life and more likely to have young children than older people with dementia.

### Strengths and Limitations

To our knowledge, there is little research on family carers’ experiences with the supporter service for people with YOD. Our study contributes knowledge that enables the development of more tailored and better individualized health care services for people with YOD and their families. These individuals have unique requirements for support related to the earlier onset of dementia in the life course. We argue that our study may contribute knowledge about the supporter service that is also transferable to family carers of older people with dementia who need individualized assistance, support, and relief.

The limitations of this study include the rather small sample size and the fact that the participants were interviewed only once. Future research should follow families and supporters over a longer period of time, as dementia progresses.

## 5. Conclusions

This study demonstrates that a well-organized supporter service can be of great value both to family carers and to people with YOD. The supporter service should be expanded and be more systematically coordinated with the formal health system. This service requires good communication and dialogue with the people with dementia and their carers, sensitivity to their needs, and the ability to be flexible and supportive in both contact and content. The supporter service is of particular value given its capacity to provide personal, individualized contact, support the self, and provide a sense of normality. Receiving this type of service may sustain a sense of coherence in life as dementia transforms the existence of people with YOD.

## Figures and Tables

**Table 1:** Characteristics of the primary family carers of people with young-onset dementia (YOD) and the person with YOD.

Family Carers			People with YOD				
Relationship (Age in Years)	Cohabitation Yes/No	Formal Services	* Gender (Age in Years)	Time Since Diagnosis (Years)	Symptoms Before Diagnosis (Years)	Supporter (Years)	Other Services
Wife (56)	Yes	Support group for carers ^e^	M (57)	1.5	6	3 (2)	Day care center ^b,c^
Husband (62)	Yes	Municipality support	F (-)	4	7	1 (-) ^a^	Day care center ^b,c,e^
Sister (59)	No	Municipality support/support group ^e^	F (54)	1.5	5–6	1.5 (1) ^a^	Day care center ^e^
Son (38)	No	Hospital and municipality support	F (-)	1.5	6	1 (1)	Home care service
Daughter (29)	No	Support group for carers ^e^	M (62)	3.5	-	2 (1)	Day care center ^c^
Wife (54)	Yes	Municipality support/support group ^e^	M (61)	0.6	7	2.5	Nursing home
Partner (62)	Yes	Hospital and municipality support	F (60)	3	5	1 (1) ^a^	None
Husband (57)	No	Municipality support	F (55)	0.6	2–3	1 (1) ^a^	Nursing home ^d^
Daughter (43)	No	Municipality support	F (64)	4	5–6	4 (2)	Nursing home ^d,e^
Husband (69)	Yes	Hospital support/support group	F (63)	4	16	3 (2) ^a^	None
Daughter (43)	No	Municipality support/support group	f (65)	5	5–6	3 (3) ^a^	Day care
Husband (66)	Yes	Hospital support	F (68)	6	7–8	1 (1)	Day care
Brother (57)	No	Municipality support	M (61)	2	5	1.5 (3)	Nursing home ^d,e^
Partner (65)	No	Municipality support/support group	F (62)	4	5	1 (2)	None
Daughter (42)	No	Municipality support	M (70)	7	8	2 (-)	Nursing home
Husband (62)	Yes	Hospital support	F (58)	3	2	1 (1) ^a^	None

* M = male, F = female; ^a^ The supporter is a family member or a friend of the family; ^b^ Receives home-care services; ^c^ Receives short-term institutionalization services; ^d^ Receives supporter services; ^e^ Specialized service for people with YOD.

## References

[1] Engedal K., Haugen P.K. (2009). Demens Fakta og Utfordringer [Dementia, Facts and Demands].

[2] Van Vliet D., de Vugt M.E., Bakker C., Koopmans R.T., Verhey F.R. (2010). Impact of early onset dementia on carers: A review. Int. J. Geriatr. Psychiatry.

[3] Rosness T.A., Mjørud M., Engedal K. (2011). Quality of life and depression in carers of patients with early onset dementia. Aging Ment. Health.

[4] Johannessen A., Möller A. (2013). Experiences of people living with early-onset dementia in everyday life: A qualitative study. Dementia.

[5] Croog S.H., Burleson J.A., Sudilovsky A., Baume R.M. (2006). Spouse caregivers of Alzheimer patients: Problem responses to caregiver burden. Aging Ment. Health.

[6] Ikejima C., Yasuno F., Mizukami K., Sasaki M., Tanimukai S., Asada T. (2009). Prevalences and causes of early-onset dementia in Japan: A population based study. Stroke.

[7] Zhu X.C., Tan L., Wang H.F., Jiang T., Cao L., Wang C., Wang J., Tan C.-C., Meng X.-F., Yu J.-T. (2015). Rate of early onset Alzheimer’s disease: A systematic review and meta-analysis. Ann. Transl. Med..

[8] Baptista M.A.T., Santos R.L., Kimura N., Lacerda I.B., Johannessen A., Barca M.L., Engedal K., Dourado M.C.N. (2016). Quality of life in young onset dementia: An updated systematic review. Trends Psychiatry Psychother..

[9] Barca M.L., Thorsen K., Engedal K., Haugen P.K., Johannessen A. (2014). Nobody asked me how I felt: Experiences of adult children of people with young-onset dementia. Int. Psychogeriatr..

[10] Ducharme F., Kergoat M.-J., Antoine P., Pasquier F., Coulombe R. (2013). The unique experiences of spouses in early-onset dementia. Am. J. Alzheimer’s Dis. Other Dement..

[11] Haugen P.K. (2012). Demens før 65 år—Fakta, Utfordringer og Anbefalinger [Dementia before 65 Years].

[12] Johannessen A. (2012). Dementia and Public Health—With Focus on Access to Society.

[13] Kimura N.R., Maffioletti V.L., Santos R.L., Baptista M.A., Dourado M.C. (2015). Psychosocial impact of early onset dementia among caregivers. Trends Psychiatry Psychother..

[14] Johannessen A., Engedal K., Thorsen K. (2016). Coping efforts and resilience among adult children growing up with a parent with young-onset dementia: A qualitative follow-up study. Int. J. Qual. Stud. Health Wellbeing.

[15] Kaiser S., Panegyres P.K. (2007). The psychosocial impact of young onset dementia on spouses. Am. J. Alzheimer’s Dis. Other Dement..

[16] Johannessen A., Helvik A.-S., Engedal K., Thorsen K. (2016). Experiences and needs of spouses of people with young-onset frontotemporal lobe dementia during the progression of the disease. Scand. J. Caring Sci..

[17] Dementia Plan 2020, Oslo, Norway. https://www.regjeringen.no/no/dokumenter/demensplan-2020/id2465117/.

[18] Helse og Omsorgsdepartementet (2011). Lov om Kommunale Helse- og Omsorgstjenester [Act of Community Health and Care Services]. https://lovdata.no/dokument/NL/lov/2011-06-24-30.

[19] Gjøra L., Eek A., Kirkevold Ø. (2014). Nasjonal Kartlegging av Tilbudet Til Personer Med Demens (National Survey of Services to People with Dementia).

[20] Johannessen A., Hallberg U., Möller A. (2013). Motivating and discouraging factors with being a support contact in the dementia care sector: A grounded theory study. Scand. J. Disabil. Res..

[21] Johannessen A., Möller A. (2012). Why do administrators employ or not employ support contacts? A Norwegian qualitative study. Nord. J. Soc. Res..

[22] Graneheim U., Lundman B. (2004). Qualitative content analysis in nursing research: Concepts, procedures and measures to achieve trustworthiness. Nurse Educ. Today.

[23] Kvale S. (1983). The qualitative research interview—A phenomenological and a hermeneutical mode of understanding. J. Phenomenol. Psychol..

[24] Kvale S., Kvale S. (1989). To validate is to question. Issues of Validity in Qualitative Research.

[25] World Medical Association Declaration of Helsinki. Proceedings of the 64th WMA General Assembly.

[26] O’Connell B., Hawkins M., Ostaszkiewicz J., Millar L. (2012). Carers’ perspective of repite care in Australia: An evaluative study. Contemp. Nurse.

[27] Atchley R.C. (1989). A continuity theory of normal aging. Gerontologist.

[28] Donnellan W.J., Benett K.M., Soulsby L.K. (2016). What are the factors that facilitate or hinder recilience in older spousal dementia carers? A qualitative study. Aging Health.

[29] Amichai-Hamburger Y., Kingsbury M., Schneider B. (2013). Friendship: An old concept with a new meaning?. Comput. Hum. Behav..

[30] Nicolaisen M., Thorsen T. (2016). What are friends for? Friendships and loneliness over the lifespan—From 18 to 79 years. Int. J. Aging Hum. Dev..

[31] Fischer C.S. (1982). To Dwell among Friends.

[32] Matthews S. (1986). Friendships through the Life Course: Oral Biographies in Old Age.

[33] Shanley C. (2016). Developing more flexible approaches to respite for people living with dementia and their carers. Am. J. Alzheimer’s Dis. Older Dement..

[34] Tolhurst E., Bhattacharyya S., Kingston P. (2014). Young onset dementia: The impact of emergent age-based factors upon personhood. Dementia.

[35] Antonovsky A.A. (1984). A call for a new question—salutogenesis—and a proposed answer—The sense of coherence. J. Prev. Psychiatry.

[36] Söderhamn U., Landmark B., Aasgaard L., Eide H., Soderhamn O. (2012). Volunteering in dementia care—A Norwegian phenomenological study. J. Multidiscip. Healthc..

[37] Van der Ploeg E.S., Walker H., O’Connor D.W. (2014). The feasibility of volunteers facilitating personalized activities for nursing home residents with dementia agitation. Geriatr. Nurs..

